# Measuring Teachers’ Social-Emotional Competence: Development and Validation of a Situational Judgment Test

**DOI:** 10.3389/fpsyg.2020.00892

**Published:** 2020-05-29

**Authors:** Karen Aldrup, Bastian Carstensen, Michaela M. Köller, Uta Klusmann

**Affiliations:** ^1^Department of Educational Research and Educational Psychology, IPN – Leibniz Insitute for Science and Mathematics Education, Kiel, Germany; ^2^Institute for Psychology of Learning and Instruction, Kiel University, Kiel, Germany

**Keywords:** social-emotional competence, emotion regulation, situational judgment test, teacher-student relationship, teacher well-being

## Abstract

Teachers’ social-emotional competence is considered important in order to master the social and emotional challenges inherent in their profession and to build positive teacher-student relationships. In turn, this is key to both teachers’ occupational well-being and positive student development. Nonetheless, an instrument assessing the profession-specific knowledge and skills that teachers need to master the social and emotional demands in the classroom is still lacking. Therefore, we developed the Test of Regulation in and Understanding of Social Situations in Teaching (TRUST), which is a theory-based situational judgment test measuring teachers’ knowledge about strategies for emotion regulation and relationship management in emotionally and socially challenging situations with students. Results from three studies (*N* = 166 in-service teachers, *N* = 73 in-service teachers, *N* = 107 pre-service teachers) showed satisfactory internal consistency for both the emotion regulation and relationship management subtests. Furthermore, confirmatory factor analyses supported the differentiation between the two facets of social-emotional competence. Regarding convergent validity, results from Study 3 revealed a positive association between the profession-specific TRUST and pre-service teachers’ general emotional intelligence. Furthermore, small to moderate correlations with the Big Five personality traits provided evidence for the discriminant validity of TRUST. In Studies 1 and 2, we found evidence for a correlation with external criteria, that is, teachers with higher test scores reported providing more emotional support for students and having better teacher-student relationships. For teachers’ occupational well-being, we found a link with symptoms of depersonalization and job satisfaction, but none for emotional exhaustion. We will discuss the use of TRUST in research, for the evaluation of interventions, in teacher education, and professional development and will illustrate ideas for enhancing the tool.

## Introduction

Social interactions between teachers and students and the quality of their relationship are vital for students’ cognitive, social, and affective-motivational development (Cornelius-White, 2007; Roorda et al., 2011; Kunter et al., 2013; Hamre et al., 2014; Aldrup et al., 2018a). However, when students disobey rules, are noisy and disturb instruction, are disengaged or not focused, teachers often experience negative emotions and struggle to maintain positive relationships with them (Hargreaves, 2000; Frenzel et al., 2009; McGrath and van Bergen, 2015; Nurmi and Kiuru, 2015; Aldrup et al., 2018b). In the long run, teachers’ feelings of anger or anxiety and the inability to effectively interact and build connections with students are associated with lower occupational well-being (Schutz and Zembylas, 2009; Klassen et al., 2012; Dicke et al., 2015; Aldrup et al., 2017, 2018b). Moreover, teachers who feel depleted of their emotional resources have been found to be less sensitive and to provide less emotional support in their interaction with students and their classes had lower motivation and achievement (Shen et al., 2015; Arens and Morin, 2016; Klusmann et al., 2016; Koenen et al., 2018). Thus, identifying teacher characteristics that support them in dealing with their own emotions and in promoting positive teacher-student relationships—even in challenging social interactions with students—is highly relevant for both student development and teachers’ occupational well-being.

In this regard, scholars have emphasized the central role of teachers’ social-emotional competence for over a decade (Brackett and Katulak, 2006; Jennings and Greenberg, 2009). However, due to a lack of objective assessment tools specifically designed to cover teachers’ profession-specific demands, it is still difficult to empirically investigate which types of knowledge and skills teachers should acquire, for example in teacher education and professional development programs, in order to master the social and emotional challenges in the school-context. By developing the Test of Regulation in and Understanding of Social Situations in Teaching (TRUST), a theory-driven situational judgment test, we aimed to provide a solution to this problem. This contribution describes the development process of the TRUST and presents results from three empirical studies (*N* = 166 in-service teachers, *N* = 73 in-service teachers, *N* = 107 pre-service teachers), investigating its reliability and construct validity as well as associations with the quality of teacher-student relationships and teacher well-being.

### The Concept of Social-Emotional Competence

Social-emotional competence refers to a person’s knowledge, skills, and motivation required to master social and emotional situations (Elias et al., 1997; also see Weinert, 2001). In defining the prerequisites that allow people to succeed in social and emotional situations more precisely, different theoretical perspectives, including the fields of emotional intelligence (Boyatzis et al., 2000; Mayer et al., 2008), social-emotional learning (Zins et al., 2004), and social competence research (Rose-Krasnor, 1997; Nangle et al., 2010) largely agree. Namely, these strands of research mention awareness of one’s own emotions and emotion regulation skills on the one hand, and the awareness of other people’s emotions and relationship management skills on the other hand. Thereby, a hierarchical order of these skills is assumed where awareness of one’s own and other people’s emotions are considered precursors of the most advanced skills of emotion regulation and relationship management (Mayer and Salovey, 1997; Joseph and Newman, 2010). Consequently, to succeed in the complex social and emotional demands of the teaching profession, emotion regulation and relationship management are inevitable, whereas awareness of own and other emotions alone are not sufficient. Therefore, we decided to focus on the measurement of emotion regulation and relationship management skills in developing the TRUST.

#### Teachers’ Emotion Regulation

Emotion regulation “refers to the (conscious and unconscious) processes by which individuals influence which emotions they have, when they have them, and how they experience and express these emotions” (Gross, 1998, p. 275). Gross (1998) suggested that people use various emotion regulation strategies. Among the most frequently applied emotion regulation strategies—in the general population, but also for regulating teachers’ emotions in the classroom—are problem solving, cognitive reappraisal, activity and social support, avoidance, suppression, and rumination (Sutton, 2004; Burić et al., 2017; Izadpanah et al., 2017; Taxer and Gross, 2018). These strategies are considered differentially effective for maintaining affective well-being (Sheppes and Gross, 2012). Empirical research with teachers showed that problem solving and cognitive reappraisal are associated with higher well-being, whereas teachers stating they frequently hide negative emotions have lower well-being (Aldao et al., 2010; Tsouloupas et al., 2010; Taxer and Frenzel, 2015; Lee et al., 2016; Burić et al., 2017; Yin et al., 2018). Furthermore, students perceive their teacher’s negative emotions even when they try not to express them, which likely interferes with the quality of teacher-student interactions (Sutton and Wheatley, 2003; Jiang et al., 2016).

#### Teachers’ Relationship Management

In general, relationship management includes skills regarding communication, the ability to notice when others need help and to offer appropriate support, conflict management, negotiation, and setting limits—hence, the ability to respond to other people’s needs while asserting one’s own goals is considered important to build positive relationships (Rose-Krasnor, 1997; Zins et al., 2004; Nangle et al., 2010). In the teaching profession, these skills are reflected in prominent models of instructional quality such as the CLASS framework (Hamre and Pianta, 2007), which is a theory-driven and well-established approach to describe the domains of teacher-student interactions that are important for students’ cognitive and psychosocial development (Allen et al., 2013; Downer et al., 2014; Hafen et al., 2015). On the one hand, the *emotional support* domain includes respectful, encouraging, and warm communication and the provision of individual help when students face emotional and academic problems, or when there are conflicts among peers (Pianta et al., 2012; Strati et al., 2017). On the other hand, skills in negotiation and setting limits are central for effective *behavior management*, that is, the teachers’ ability to maximize time-on-task and create a calm learning environment by stating clear behavioral expectations and rules, monitoring student behavior, and using subtle cues to redirect misbehavior (Emmer and Stough, 2001; Evertson and Weinstein, 2006).

### Assessment of Teachers’ Social-Emotional Competence

Several self-report questionnaires are available to assess emotion regulation and relationship management skills in adults. For example, the Emotion Regulation Questionnaire (ERQ; Gross and John, 2003) asks participants to rate how often they apply reappraisal and suppression, and the Interpersonal Competence Questionnaire (ICQ; Buhrmester et al., 1988) assesses the degree to which people view themselves as able to initiate relationships, to seek and provide emotional support, to assert themselves and resolve conflicts. Combining scales for emotion regulation and relationship management skills, the Trait Emotional Intelligence Questionnaire (TEIQue; Petrides, 2009) includes, for example, the degree to which people perceive themselves as capable of controlling their own emotions, influencing other people’s feelings, asserting themselves, and building positive relationships (also see Freudenthaler et al., 2008). Regarding the validity of self-report scales of social-emotional competence, prior research has established a relationship with self-reported social functioning and well-being (e.g., Freudenthaler et al., 2008; Kanning, 2006; Lee et al., 2016; Burić et al., 2017). However, empirical studies call into question whether a person’s subjective perspective on their social-emotional competence relates to other people’s evaluations of their social behavior. For instance, Brackett et al. (2006) showed no relationship between teachers’ self-reported emotional intelligence and the extent to which others perceived them as friendly and socially engaged. Furthermore, associations between teacher- and student-reported emotional support are rather low, indicating that teachers may not be able to accurately evaluate the quality of interpersonal behavior in the classroom (e.g., Hughes and Kwok, 2007; Downer et al., 2014; Wagner et al., 2016; Aldrup et al., 2018a). In addition, large correlations between self-report measures of social-emotional competence and personality traits raise the question of their conceptual distinctness (e.g., Brackett and Mayer, 2003; Freudenthaler et al., 2008; Joseph et al., 2015). Finally, the use of self-report questionnaires poses the risk of inflated correlations due to common method bias when participants report on their social-emotional competence and on their well-being or other outcomes at the same time (Podsakoff et al., 2003). Objective tests provide a solution to these problems.

For instance, the Mayer-Salovey-Caruso Emotional Intelligence Test (MSCEIT; Mayer et al., 2002), the Situational Test of Emotional Understanding (STEU; MacCann and Roberts, 2008), and the Situational Test of Emotional Management (STEM; MacCann and Roberts, 2008) measure a person’s ability to correctly recognize emotions and evaluate the effectiveness of different emotion regulation strategies in specific situations, which are sometimes social. Supporting the validity of these instruments, prior studies have found a positive association with well-being, friends’ ratings of relationship quality, and supervisor ratings of job performance in high emotional labor professions (Lopes et al., 2004; Joseph and Newman, 2010; Fernández-Berrocal and Extremera, 2016; for an overview see Mayer et al., 2008). In the teaching profession, higher scores in the MSCEIT have been linked to more job satisfaction and positive affect, as well as to lower burnout (Brackett et al., 2010). However, Corcoran and Tormey (2013) did not find the expected positive correlation between scores in the MSCEIT subtests and student teachers’ performance rankings in their teaching practicum. Yet, in addition to social and emotional aspects, such as the quality of teacher-student relationships and appropriateness of pedagogic strategies, job performance also included facets such as planning, selection of materials, or pedagogical content knowledge. Thus, on the one hand, the unexpected finding could be because performance was not restricted to the social-emotional domain. On the other hand, emotional intelligence measured at a very general level might be less predictive of performance in specific contexts (Weinert, 2001; Monnier, 2015). In this regard, it is important to acknowledge the unique, asymmetric nature of teacher-student interactions that potentially requires profession-specific knowledge and skills for teachers to succeed (Pianta, 1999; Kunter et al., 2013). In addition, profession-specific display rules for emotions may affect the ways in which teachers deal with their affective experiences (Sutton, 2004). Further underpinning the idea that a context-specific assessment of social-emotional competence is needed, Smith et al. (2018) found that an intervention targeting the theories of emotions of adolescents in the school context was associated with greater school-related well-being, yet their general well-being remained unchanged.

One of the few approaches to measure single components of teachers’ social-emotional competence both objectively and profession-specifically was the development of tests for teachers’ general pedagogical-psychological knowledge. Alongside with aspects such as knowledge about structuring lessons and classroom assessment, these tests (Voss et al., 2011; König and Pflanzl, 2016) measure knowledge that should make teachers more aware of students’ needs and enable successful social interactions in the classroom (i.e., knowledge about student heterogeneity, strategies for classroom management, and motivating students). Prior studies revealed that teachers with higher general pedagogical-psychological knowledge had better teacher-student relationships, greater awareness of students’ comprehension problems, and fewer classroom disturbances—as reported by students (Voss et al., 2011; König and Pflanzl, 2016). However, current tests of teachers’ general pedagogical-psychological knowledge largely neglect emotional aspects of teacher-student interactions. That is, they neither assess whether teachers know how to support their students emotionally, nor whether teachers are able to deal with their own emotions while interacting with students. Therefore, our goal was to develop an objective and profession-specific assessment that covers these aspects as well.

## The Present Contribution

From a theoretical perspective, it seems evident that teachers require social-emotional competence for quality teacher-student relationships and teacher well-being (Brackett et al., 2006; Jennings and Greenberg, 2009). However, there is still limited empirical research testing the idea that teachers’ knowledge and skills regarding emotion regulation and relationship management—two central components of social-emotional competence—are associated with positive outcomes for both students and teachers. From our perspective, the lack of valid, profession-specific tools for assessing teachers’ social-emotional competence forms a clear obstacle in the research field. Therefore, we developed the theory-driven situational judgment test TRUST. The goal was to provide a tool, not only for research in teachers’ social-emotional competence, but also for reflection and learning in professional development and teacher education.

The test confronts teachers with emotionally and socially challenging situations with students and asks them to rate the effectiveness of different response choices for either regulating their own emotions or for establishing and maintaining a positive teacher-student relationship. The development of a profession-specific situational judgment test holds several advantages. First, rather than a self-report questionnaire we provide an objective test, which is more likely to validly predict social behavior in the classroom and is less prone to common method bias (for a discussion of this issue also see Brackett et al., 2006). Second, situational judgment tests are a widespread and valid approach from personnel psychology that has been successfully used to measure procedural knowledge and to predict future job performance (McDaniel et al., 2001; McDaniel et al., 2007; Lievens and Motowidlo, 2016). Recently, Klassen et al. (2020) imposingly demonstrated the potential of situational judgment tests for teacher selection. In contrast to the TRUST, which is an in-depth measure of social-emotional competence, they developed a very comprehensive tool, which assesses an aggregate of conscientiousness, organization, growth mindset, adaptability, empathy, and emotion regulation. Klassen et al. (2020) showed that their test predicted performance in an assessment center for teacher candidates. Third, the profession-specificity of TRUST makes it distinct from similar tools for use in the general population (e.g., MSCEIT; Mayer et al., 2002). In taking a profession-specific approach, we acknowledge that profession-specific knowledge is needed to succeed in teacher-student interactions, as well as the fact that profession-specific display rules may affect the ways in which teachers express their emotions (Sutton, 2004; Kunter et al., 2013).

In the present contribution, we present evidence from three empirical studies investigating the reliability and validity of the TRUST, based on two samples of in-service teachers and one sample of pre-service teachers. This allowed us to examine whether the measure is reliable in different samples and applicable at different stages of professionalization. First, we analyzed the item-functioning of the TRUST in one in-service teacher sample and eliminated items with poor performance (i.e., low item-total correlation). In addition, we examined the reliability of the resulting test version and additionally tested whether similar item characteristic and reliability resulted in the two other samples.

Second, we investigated the factorial validity. We expected to find two factors—emotion regulation and relationship management skills—that were distinct but correlated because they are both part of the larger construct of social-emotional competence (Zins et al., 2004; Mayer et al., 2008). Furthermore, we investigated whether the factor structure was comparable across different experience levels of participants, that is, whether there was measurement invariance across the in-service and pre-service teacher samples.

Third, we examined convergent validity with emotional intelligence and discriminant validity regarding the Big Five personality traits in the sample of pre-service teachers to test whether TRUST was associated, yet distinguishable from related concepts. Due to the theoretical overlap, we expected a moderate association between the TRUST and established measures of emotional intelligence for use in the general population. More specifically, we assumed to find particularly close associations between the TRUST *emotion regulation* and the MSCEIT *emotion management* subtests and between the TRUST *relationship management* and the MSCEIT *emotional relationships* subtests. Nonetheless, we did not anticipate a large correlation because MSCEIT is a general tool, whereas TRUST is likely to require profession-specific knowledge about how to act in teacher-student interactions. Regarding personality, positive, not larger than moderate correlations with agreeableness, extraversion, conscientiousness, openness, and emotional stability appeared plausible and in line with prior theoretical assumptions and research (O’Brien and DeLongis, 1996; Gross and John, 2003; Schulte et al., 2004).

Fourth, we examined criterion validity by testing whether TRUST predicted better teacher-student interactions and higher occupational well-being among in-service teachers. These hypotheses were based on the theoretical idea that social-emotional competence should enable teachers to master the manifold social and emotional challenges of their profession, for instance, dealing with student misbehavior, disengagement, learning difficulties, or negative teacher-student relationships (Elias et al., 1997; Rose-Krasnor, 1997; Gross and John, 2003; Brackett and Katulak, 2006; Jennings and Greenberg, 2009). Furthermore, there is initial empirical evidence showing that aspects of social-emotional competence or theoretically overlapping constructs, such as general pedagogical-psychological knowledge, are associated with teacher well-being and the quality of teacher-student interactions (e.g., Voss et al., 2011; Taxer and Frenzel, 2015; Jennings et al., 2017).

## Materials and Methods

### Test of Regulation in and Understanding of Social Situations in Teaching

We constructed the TRUST for measuring two central facets of teachers’ social-emotional competence—emotion regulation and relationship management skills (Mayer et al., 2002; Zins et al., 2004). The *emotion regulation* subtest assesses the teacher’s ability to change their emotional experiences and expressions when facing emotionally challenging teacher-student interactions. The *relationship management* subtest measures the teacher’s ability to build positive teacher-student relationships and maintain them when confronted with difficulties.

#### Structure of the Test

**FIGURE 1:**
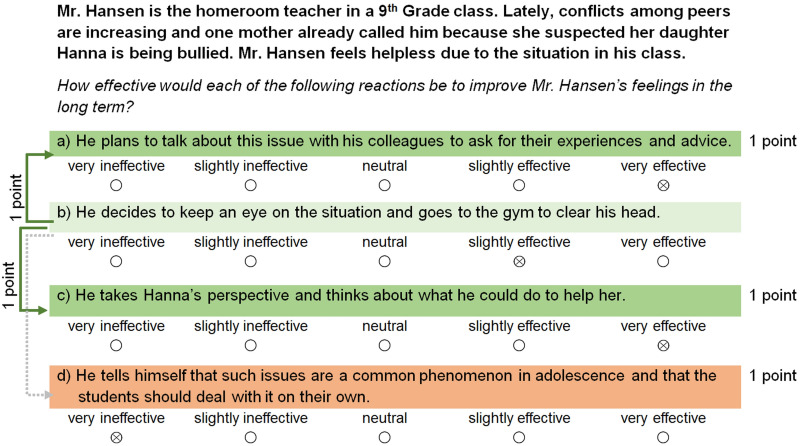
Example scenario from the *emotion regulation* subtest including the four potential reactions and their effectiveness. The exemplary respondent received one point for rating each of the very (in-)effective strategies (dark green/orange) correctly. Reaction (b) is an ambiguous strategy with a tendency to being effective (light green). The solid line represents pairwise comparisons with strategies that are adjacent regarding their effectiveness; the respondent received one point for correctly rating (b) at least one unit worse than (a) and (c). The dotted line implies a pairwise comparison with a more distant strategy. In this case, the participant correctly rated (b) at least two units better than (d). However, this pairwise comparison was excluded from the final test version due to a low item-total correlation.

**FIGURE 2:**
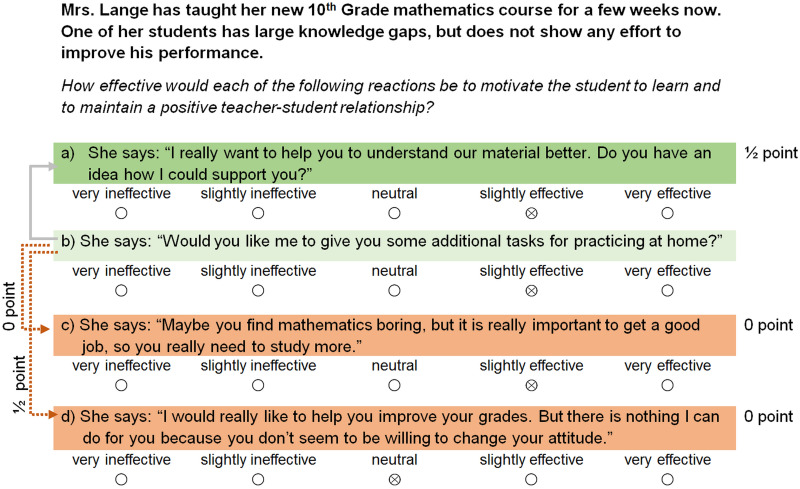
Example scenario from the *relationship management* subtest including the four potential reactions and their effectiveness. The exemplary respondent slightly underestimated the (in-)effectiveness of the very effective option (a) (dark green) and received only partial points. Furthermore, the effectiveness of the very ineffective strategies (c) and (d) (orange) was clearly overestimated resulting in no points for these items. Reaction (b) is an ambiguous strategy with a tendency to being effective (light green). The solid line represents a pairwise comparison with a strategy that is adjacent regarding its effectiveness; the respondent would have received half a point for rating (b) and (a) alike. However, this pairwise comparison was excluded from the final test version due to a low item-total correlation. The dotted line implies pairwise comparisons with more distant strategies. In this case, the participant rated (b) only one unit better than (d), which resulted in half a point for this pairwise comparison. Furthermore, he or she did not differentiate between (b) and (c), which resulted in zero points for this pairwise comparison.

Similar to established measures of emotional intelligence, such as MSCEIT (Mayer et al., 2002) and STEM (MacCann and Roberts, 2008), in both subtests, teachers first read a short scenario that is emotionally relevant for the teacher (emotion regulation, eight scenarios) or concerns the quality of the teacher-student relationship (relationship management, nine scenarios). Subsequently, we present four potential reactions and the teachers are asked to rate each alternative regarding their effectiveness for making themselves feel better (emotion regulation, 32 items) or building/maintaining a positive teacher-student relationship (relationship management, 36 items) on a five-point scale ranging from 1 = *very ineffective* over 3 = *neutral* to 5 = *very effective*. We will be pleased to share the full set of items with interested researchers upon request and present an example scenario of each subtest in Figures 1, 2.

#### Process of Test Development

**FIGURE 3:**
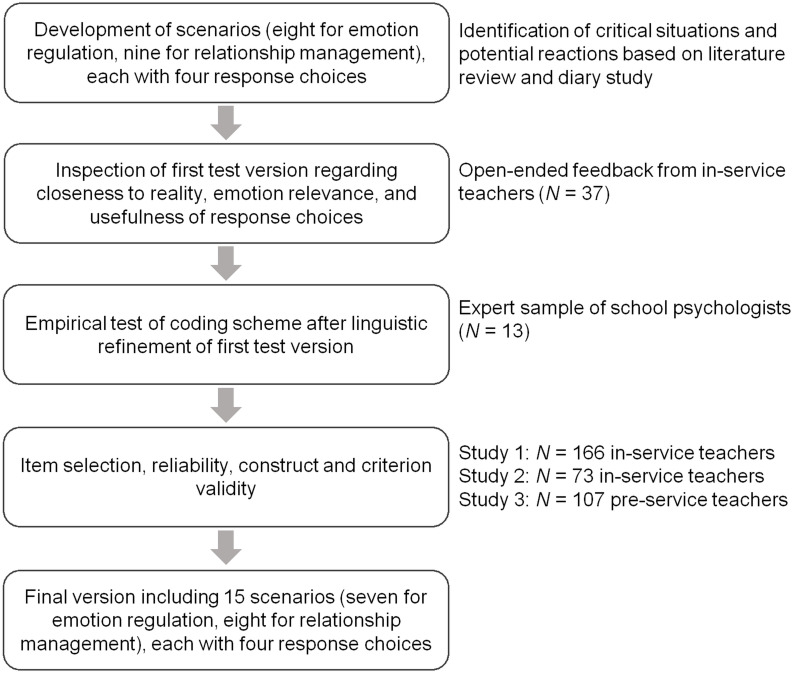
Overview of the process of test development with the key steps in the left part of the flow diagram and the methods applied in each step in the right part.

In developing the test, the first step was to identify situations relevant to teachers’ emotions and to the teacher-student relationship (see Figure 3 for an overview of the whole development process). To increase content and face validity, our goal was to include a broad range of interactions between teachers and their students. Hence, we examined studies on teachers’ daily work-related experiences, teacher emotions, and teacher-student relationships (e.g., Hargreaves, 2000; Schmidt et al., 2017). The situations that we identified included four broader themes, also in line with the model of teacher emotions by Frenzel (2014): students’ motivation (e.g., lack of behavioral engagement or concentration), students’ social-emotional behavior (e.g., violation of rules, conflicts among peers), student achievement (e.g., learning problems), and the teacher-student relationship *per se* (e.g., relationship building at the beginning of school year, student hostility). Furthermore, the situations were changeable to diverging degrees and addressed short- and long-term concerns.

Based on theory and prior research, we then developed four potential reactions that ranged from *very effective* to *very ineffective* for successful emotion regulation and relationship management in a given situation. In the subtest of *emotion regulation*, each reaction reflected a specific emotion regulation strategy (Gross, 1998; Izadpanah et al., 2017): cognitive reappraisal (nine of the potential reactions), problem solving (eight of the potential reactions), seeking activity or social support (eight of the potential reactions), suppression (three of the potential reactions), rumination (two of the potential reactions), avoidance (one of the potential reactions), and expression (one of the potential reactions). In developing response choices for the *relationship management* subtest, we were guided by the CLASS framework and constructed the potential reactions to reflect diverging degrees of emotional support and behavior management (Hamre and Pianta, 2007; Pianta et al., 2012). That is, each reaction combined differentially effective ways to establish a positive climate (e.g., respond friendly versus display irritability) and to address students’ academic and social-emotional needs or behavioral issues.

To evaluate the test’s face validity, we conducted a preliminary study with *N* = 37 in-service teachers. Participants were asked for feedback in an open-ended format after reading each scenario and the corresponding response choices. Their feedback showed that the scenarios were realistic and emotionally relevant and the response choices useful.

#### Scoring of the Test

There are different strategies for scoring situational judgment tests, each with specific advantages and drawbacks (for an overview see Bergman et al., 2006). The most common approach is to ask experts to respond to the test and award more points the greater the consensus between participants’ and experts’ ratings (e.g., Mayer et al., 2002; Klassen et al., 2020). However, MacCann and Roberts (2008) suggested theory-based scoring as a valuable alternative because it allows for a better understanding of the captured construct as it is comprehensible *why* a specific strategy is effective or ineffective. Therefore, we developed a theory-based coding scheme to make scores interpretable against the background of the models that guided test development—the process model of emotion regulation (Gross, 1998) and the CLASS framework of effective teacher-student interactions (Hamre and Pianta, 2007). Based on these models, we organized the potential reactions into three broad groups: *very effective, very ineffective*, and *ambiguous.*

The *very effective* strategies were clearly conducive, and the *very ineffective* strategies were clearly detrimental to optimal emotional and social functioning. Participants received one point for correctly scoring a very effective strategy as 5 = *very effective*, and half a point for rating it as 4 = *slightly effective.* Similarly, scoring a very ineffective strategy as 1 = *very ineffective* yielded one point and rating it as 2 = *slightly ineffective* resulted in half a point.

Ambiguous strategies were those for which we considered responses at 2 = *slightly ineffective*, 3 = *neutral*, and 4 = *slightly effective* correct. In other words, these strategies were ineffective, but at least not harmful, or effective only to a limited degree. First, we aimed to score the ambiguous strategies analogous to the very (in-)effective strategies, but this resulted in poor item functioning. Because a clear-cut correct answer could not be determined for the ambiguous strategies, we established a more complex coding procedure, in which we awarded points if respondents correctly differentiated the ambiguous from the very (in-)effective strategies (for a similar approach see Artelt et al., 2009; Gold and Holodynski, 2015).

More precisely, because we considered it quite difficult to differentiate between strategies that were similar in their effectiveness, respondents received half a point for rating these *adjacent strategies* alike and one point for correctly distinguishing between the two. *Adjacent strategies* were (a) ambiguous strategies with a tendency to being effective versus very effective strategies, and (b) ambiguous strategies with a tendency to being ineffective versus very ineffective strategies. In contrast, we wanted respondents to differentiate clearly between *distant strategies*, that is, (c) ambiguous strategies with a tendency to being effective versus very ineffective strategies, or (d) ambiguous strategies with a tendency to being ineffective versus very effective strategies. Hence, respondents received half a point if the effectiveness ratings differed by one unit and one point if the effectiveness ratings differed by at least two units. We illustrate the scoring procedure based on two examples in Figures 1, 2. Finally, the total score for each subtest was derived by adding the number of points for the individual very effective and very ineffective strategies on the one hand, and for the pairwise comparisons of the ambiguous strategies with the very (in-)effective strategies on the other hand.

To provide empirical support for our theory-based coding scheme, we asked *N* = 13 school psychologists to complete the test. At least half of them chose the exact correct answer for 79% of the items and pairwise comparisons in the *emotion regulation* subtest and for 67% of the items and pairwise comparisons in the *relationship management* subtest. The experts reached 78.85% of the possible points in the *emotion regulation* subtest and 71.67% of the possible points in the *relationship management* subtest, indicating substantial overlap between our coding scheme and expert views (for detailed results see Supplementary Material).

### Samples and Procedures

We collected data from two samples of in-service teachers (Study 1, Study 2) and one sample of pre-service teachers (Study 3). Based on recommendations in the methodological literature for minimum sample sizes for conducting factor analyses, we aimed to recruit at least 100 participants per sample (Worthington and Whittaker, 2016). Participation was voluntary and we obtained informed, written consent from all individuals, and carefully followed the ethical principles of the American Psychological Association (2017).

#### Study 1

Study 1 was part of a larger research project examining teachers’ daily well-being and experiences at work. The sample included *N* = 166 in-service teachers. They were, on average, *M* = 42.25 (*SD* = 8.43) years old and had 13.26 (*SD* = 7.27) years of job experience. The majority of teachers were female (72.29%) and 39.76% taught in academic track schools. We employed two strategies for recruiting participants. First, we invited teachers who had participated in a similar research project 8 years ago at the beginning of their careers (for a detailed description of the study see Schmidt et al., 2017). Second, we invited in-service teachers who were studying in the consecutive, extra-occupational master’s program “School Management and Quality Development” and asked them to inform their colleagues about the project, too. The study was conducted online and teachers filled out the TRUST, provided sociodemographic background information, and reported on their occupational well-being. In addition, participants from the extra-occupational master’s program answered questions about the perceived quality of their interactions with students, whereas teachers who had participated at the beginning of their careers responded to additional questionnaires that were not relevant for this contribution. Participation was compensated by means of a remuneration of up to 50 Euros depending on the questionnaire version.

#### Study 2

Study 2 comprised *N* = 73 in-service teachers. On average, they were *M* = 44.86 years old (*SD* = 11.05) and had *M* = 15.44 years of job experience (*SD* = 10.69). Most of the participants were female (62.39%) and taught at academic track schools (75.34%). To recruit participants, we asked principals from secondary schools in our area to forward an invitation to all teachers at their school. The invitation included background information about the study and a link allowing interested teachers access to our online survey. Similarly to Study 1, teachers first provided sociodemographic background information and then responded to the TRUST and to questions about their occupational well-being. Participants received an individual feedback report as an incentive for participation.

#### Study 3

Study 3 was conducted with a sample of *N* = 107 pre-service teachers at one university in Northern Germany. The university phase of teacher education in Germany usually spans a 3-year bachelor’s program and a 2-year master’s program. In our study, 60.75% were in the bachelor’s program and 39.25% were in the master’s program. All pre-service teachers in our sample pursued a degree for teaching in academic track schools. In contrast to vocational track schools, where teachers prepare students for vocational training, academic track schools qualify students to proceed to higher education (for a more detailed description of the German school system see Maaz et al., 2008). Participating pre-service teachers were, on average, *M* = 24.31 (*SD* = 3.17) years old and 68.22% were female. They were recruited via postings at prominent locations on campus and each participant received a remuneration of 10 Euros. Testing was conducted in a small group setting in a paper-pencil format and lasted approximately 1 hour. First, pre-service teachers provided information on their sociodemographic background. Then, they responded to the TRUST, worked on the emotional intelligence test MSCEIT, and answered a personality questionnaire.

### Instruments for Validation

#### Emotional Intelligence

We included the managing emotions facet from the German version of MSCEIT (Mayer et al., 2002; Steinmayr et al., 2011) in Study 3. The managing emotions component measures a person’s ability to regulate emotions in oneself (subtest *emotion management*) and to adequately express emotions in relationships with others (subtest *emotional relationships*). Hence, this facet was most closely aligned with the subtests of the TRUST. In the *emotion management* subtest, five scenarios are presented, and participants are subsequently asked to evaluate the effectiveness of four possible reactions for achieving or maintaining a certain emotional state on a scale from 1 = *very ineffective* to 5 = *very effective*. The *emotional relationships* subtest comprises three scenarios with three response choices each that are rated on a five-point scale (1 = *very ineffective*, 5 = *very effective*) in terms of their effectiveness for maintaining positive relationships and asserting one’s goals in social interactions. Scores on each subtest reflect the percentage of agreement between a person’s effectiveness ratings and experts’ effectiveness ratings. The reliability of the overall *managing emotions* facet was satisfactory (α = 0.74).

#### Personality

In Study 3, we measured the personality traits *agreeableness* (four items, e.g., “I give trust to others easily, believe in the good in humans,” α = 0.74), *conscientiousness* (four items, e.g., “I do a thorough job,” α = 0.70), *extraversion* (four items, e.g., “I am outgoing, sociable,” α = 0.76), *emotional stability* (four items, e.g., “I tend to get depressed, blue,” reverse coded, α = 0.68), and *openness* (five items, e.g., “I am curious about many different things,” α = 0.74) using a German short version of the Big Five Inventory (Rammstedt and John, 2005). Answers were provided on a five-point scale ranging from 1 = *completely disagree* to 5 = *completely agree.*

#### Occupational Well-Being

We aimed to measure both the positive and the negative dimensions of well-being of the in-service teachers in Study 1 and Study 2 (Diener et al., 1999). On the one hand, we measured teachers’ *job satisfaction* with a German short-version of the Job Diagnostic Survey (JDS; Hackman and Oldham, 1975; Merz, 1979), which assesses global evaluations of one’s work (five items, e.g., “Given the choice, I would definitely become a teacher again,” α = 0.83). Responses were given on a four-point scale from 1 = *strongly disagree* to 4 = *strongly agree*. On the other hand, we assessed burnout symptoms using two subscales of a short German version of the Maslach Burnout Inventory (MBI; Enzmann and Kleiber, 1989; Maslach et al., 1996). *Emotional exhaustion* is the core quality of burnout and refers to the degree to which a person feels stressed and depleted of emotional resources (four items, e.g., “I feel emotionally drained from my work,” α = 0.81). The *depersonalization* subscale assesses the extent to which teachers distance themselves from students by disregarding their individual personalities and treating them in an impersonal, callous manner (two items, “Since I am a teacher, I have become more callous towards people,” α = 0.76). Items were rated on two slightly different response scales, one ranging from 1 = *never* to 7 = *every day*, and the other ranging from 1 = *disagree* to 4 = *agree* so that we *z-*standardized teachers’ responses before calculating scale scores.

#### Teacher-Student Interaction

We assessed the quality of teacher-student interactions from the teacher perspectives in a subsample of Study 1 (*n* = 91). The teacher self-report questionnaire was developed by Baumert et al. (2008) and asked teachers to report on the degree to which they provided *emotional support* to students (nine items, e.g., “I am interested in every student’s learning progress,” α = 0.78) and were effective in terms of *behavior management* as indicated by the absence of student misbehavior (four items, e.g., “My instruction is barely disturbed,” α = 0.85). Moreover, teachers indicated whether they felt appreciated, respected, and liked by their students to reflect the quality of the *teacher-student relationship* (six items, e.g., “My students show me that they like me,” α = 0.72). The items were based on the closeness subscale of the widely applied Student-Teacher Relationship Scale (STRS; Pianta, 2001; also see Aldrup et al., 2018b). Emotional support, behavior management, and the quality of the teacher-student relationship were each rated on a four-point scale from 1 = *strongly disagree* to 4 = *strongly agree*.

### Data Analyses

As a preliminary step, item-total correlations, item difficulties, and the reliability of the TRUST were calculated using SPSS. Based on the in-service teachers in Study 1, we selected a set of items that differentiated well between participants with higher and lower social-emotional competence. Items with item-total correlations of *r*_it_ < 0.15 were excluded. We chose this comparably mild exclusion criterion for two reasons. First, the broad nature of the measured constructs and the heterogeneity of the scenarios and reactions were likely to result in lower inter-item correlations (Clark and Watson, 1995). Second, we aimed to maintain a symmetric test structure with the same amount of potential reactions for each scenario. Having selected a set of well-functioning items, we examined Cronbach’s α to check whether the reliability was acceptable. First, we investigated Cronbach’s α at the level of the individual items and pairwise comparisons. However, the pairwise comparisons lead to interdependencies among the items and pairwise comparisons within one scenario, which may result in an overestimation of Cronbach’s α. Therefore, we additionally calculated the mean score for each scenario and tested the reliability on the scenario level. Finally, we investigated whether item-total correlations and reliabilities were acceptable in another in-service teacher sample (Study 2) and in a sample of pre-service teachers (Study 3), as well.

Then, we tested the factor structure of the TRUST and its invariance across in-service and pre-service teachers. For this purpose, we conducted multiple group confirmatory factor analyses in Mplus 7 (Muthén and Muthén, (1998-2012)), using maximum likelihood estimation with robust standard errors. We followed the procedure suggested by van de Schoot et al. (2012) for testing measurement invariance across the two groups. In the first step, we estimated separate models for in-service and pre-service teachers assuming the same two-factor structure (factor 1: *emotion regulation*, factor 2: *relationship management*) in both samples, but making no presumptions about invariant factor loadings or intercepts (i.e., configural invariance). Then, we compared this model to a metric (i.e., invariant factor loadings, freely estimated intercepts) and a scalar invariant model (i.e., invariant factor loadings and intercepts). In all models, items were only allowed to load on the theoretically expected factor. Because of the large number of items and the relatively small sample size, we decided to reduce the number of parameters to be estimated by creating parcels in a first step. As for the more conservative estimation of Cronbach’s α, parcels were obtained by computing the mean score for each scenario (Little, 2013). To evaluate model fit, we considered Tucker–Lewis index (TLI) and confirmatory fit index (CFI) values ≥0.95, root mean square error of approximation (RMSEA) values ≤0.06, and standardized root mean square residual (SRMR) values ≤0.08 as indicative of good model fit (Hu and Bentler, 1999). To compare different models, we calculated Satorra-Bentler scaled χ^2^-difference tests.

Finally, we conducted correlation analyses in Mplus 7 (Muthén and Muthén, (1998-2012)) to investigate the convergent, discriminant, and criterion validities of the TRUST. This allowed us to handle the small amount of missing data in our questionnaires (0.00 to 1.27%) by using a full information maximum likelihood algorithm, as suggested in the methodological literature (Enders, 2010).

## Results

### Item Analyses and Item Selection (Studies 1–3)

As a preliminary step, we investigated the item difficulties (i.e., percentage of correct responses per item) to get a first impression of whether there was variability in teachers’ responses to the items (please note that the values in the following are based on the full set of items and pairwise comparisons and, therefore, do not fully correspond with Table 1). Across the three studies, item difficulties ranged from *P_i_* = 46.39 to *P_i_* = 93.93 for emotion regulation and from *P_i_* = 29.70 to *P_i_* = 91.67 for relationship management. On average, item difficulties in the emotion regulation subtest were *P_i_* = 67.46 in the first in-service teacher sample, *P*_i_ = 72.25 in the second in-service teacher sample, and *P_i_* = 75.14 for the pre-service teachers. In the relationship management subtest, the item difficulties were on average *P_i_* = 60.75 for the first in-service teacher sample, *P*_i_ = 64.87 for the second in-service teacher sample, and *P_i_* = 65.88 for the pre-service teachers. Hence, item difficulties were, overall, adequate and TRUST included items that were correctly answered by most respondents, as well as items that were more difficult to score.

**TABLE 1:** Item difficulties, item-total correlations, and Cronbach’s α for the TRUST subtests for the in-service teachers in Study 1 and Study 2 and the pre-service teachers in Study 3.

	Emotion regulation			Relationship management		
	Study 1 (in-service)	Study 2 (in-service)	Study 3 (pre-service)	Study 1 (in-service)	Study 2 (in-service)	Study 3 (pre-service)
**Item level^1^**						
***P*_i_**						
*M*	66.81	71.73	76.02	61.49	67.31	66.73
*Min*	50.61	47.92	51.40	29.70	46.48	38.68
*Max*	84.24	90.28	93.93	88.55	91.67	66.73
***r*_it_**						
*M*	0.33	0.32	0.26	0.30	0.31	0.25
*Min*	0.09	0.06	–0.01	0.06	–0.01	0.07
*Max*	0.50	0.65	0.46	0.55	0.65	0.42
**α**	0.83	0.82	0.74	0.82	0.84	0.76
**Scenario level^2^**						
***P*_i_**						
*M*	66.53	71.58	76.57	61.22	66.73	66.60
*Min*	60.62	68.55	67.57	49.19	51.56	56.92
*Max*	78.24	78.94	87.74	76.11	81.69	80.47
***r*_it_**						
*M*	0.44	0.38	0.29	0.40	0.43	0.30
*Min*	0.31	0.18	–0.004	0.24	0.22	0.16
*Max*	0.51	0.47	0.38	0.50	0.62	0.42
**α**	0.72	0.66	0.53	0.71	0.73	0.59

*^1^Analyses based on 33 individual items/pairwise comparisons in the emotion regulation subtest and 38 individual items/pairwise comparisons in the relationship management subtest; ^2^analyses based on the mean for all items/pairwise comparisons included in a scenario (emotion regulation: seven scenarios, relationship management: eight scenarios).*

In the next step, our goal was to check whether there were items that represented teachers’ social-emotional competence in terms of emotion regulation and relationship management only to a limited degree and that should therefore be excluded. For this purpose, we examined the corrected item-total correlations for each item and pairwise comparison with the respective subtest in the in-service teacher sample of Study 1. An item or pairwise comparison was excluded if it had an item-total correlation of *r_it_* ≤ 0.15. Based on this criterion, we excluded nine pairwise comparisons in the emotion regulation subtest. One scenario was completely excluded because the mean score for this scenario had a low correlation with the other scenarios’ mean scores (*r_it_* = 0.10). This resulted in seven scenarios for the emotion regulation subtest. For each scenario, four to six pairwise comparisons and items were included to calculate the total score. Importantly, the final version enclosed information from all of the four potential reactions presented for each scenario. The internal consistency was satisfactory both when calculated based on the individual items and pairwise comparisons (33 items and pairwise comparisons; α = 0.83) and when estimated more conservatively at the scenario level (seven scenarios; α = 0.72).

In the relationship management subtest, 11 pairwise comparisons were excluded because of low item-total correlations. Moreover, one scenario was removed completely because none of the items and pairwise comparisons met our inclusion criteria. In one scenario, we decided to keep one item and one pairwise comparison with *r_it_* < 0.15 because this did not interfere with the overall performance of the scenario and allowed us to have each potential reaction to the scenarios provide information for the computation of the final score. Altogether, this resulted in eight situations, each including four to five pairwise comparisons and items that were used for calculating the total score. The reliability was satisfactory (based on the 38 individual items and pairwise comparisons: α = 0.82; based on the eight scenarios: α = 0.71).

Finally, we drew on Study 2 and Study 3 to test whether the selected set of items and pairwise comparisons functioned satisfactorily in a different sample of in-service teachers and in a sample of pre-service teachers. Both subtests performed similarly in the second in-service teacher sample (emotion regulation: α_items_ = 0.82, α_scenarios_ = 0.66; relationship management: α_items_ = 0.84, α_scenarios_ = 0.73) and acceptably, though somewhat more poorly in the pre-service teacher sample (emotion regulation: α_items_ = 0.74, α_scenarios_ = 0.53; relationship management: α_items_ = 0.76, α_scenarios_ = 0.59). Table 1 provides an overview of the item-total correlations, item difficulties, and reliabilities for the final test version obtained in each of the three studies.

### Factorial Validity (Studies 1–3)

**FIGURE 4:**
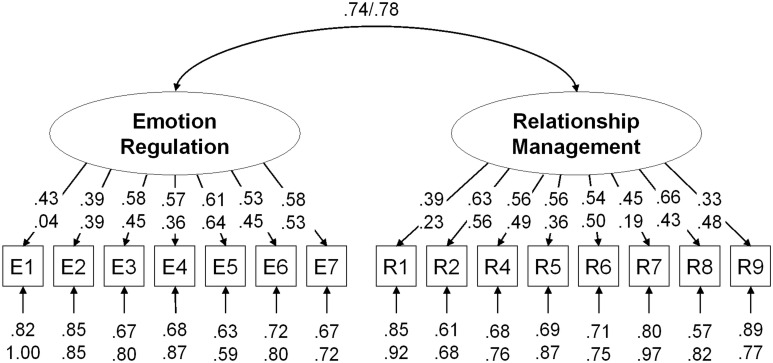
Standardized factor loadings and factor intercorrelation from the configural invariant two-factor model in confirmatory factor analyses. Results for in-service teachers are printed above results for pre-service teachers. E1–E7 = mean scores for the scenarios from the emotion regulation subtest (E8 was excluded), R1–R9 = mean scores for the scenarios from the relationship management subtest (R3 was excluded).

We conducted multiple group confirmatory factor analyses to test whether the scenarios from the two subtests reflected two underlying latent constructs (i.e., emotion regulation and relationship management skills) in both the in-service and pre-service teacher samples. We started with separate models for the in-service and pre-service teacher samples. Based on RMSEA and SRMR, the two-factor model showed acceptable fit to the data in the in-service (χ^2^ = 135.10, *df* = 89, CFI = 0.92, TLI = 0.91, RMSEA = 0.05, SRMR = 0.05) and in the pre-service teacher sample (χ^2^ = 99.75, *df* = 89, CFI = 0.93, TLI = 0.91, RMSEA = 0.03, SRMR = 0.07). As illustrated in Figure 4, standardized factor loadings ranged between 0.33 ≤ λ ≤ 0.66 (*M* = 0.52) in the in-service teacher sample and between 0.04 ≤ λ ≤ 0.64 (*M* = 0.41) in the pre-service teacher sample. Even though the latent correlation between the subtests was substantial (in-service: *r* = 0.74, pre-service: *r* = 0.78), the two-factor model was superior to a one-factor solution (in-service: χ^2^ = 171.72, *df* = 90, CFI = 0.87, TLI = 0.84, RMSEA = 0.06, SRMR = 0.06; Δχ^2^ = 36.63, Δ*df* = 1, *p* ≤ 0.001; pre-service: χ^2^ = 106.07, *df* = 90, CFI = 0.89, TLI = 0.87, RMSEA = 0.04, SRMR = 0.07; Δχ^2^ = 6.32, Δ*df* = 1, *p* ≤ 0.001). Next, we tested metric invariance by estimating a model in which the intercepts could differ between groups, whereas the factor loadings were set invariant. This model showed a similar fit as the prior model supporting metric invariance (χ^2^ = 259.09, *df* = 193, CFI = 0.91, TLI = 0.91, RMSEA = 0.04, SRMR = 0.09; Δχ^2^ = 23.60, Δ*df* = 15, *p* = 0.078). However, a scalar invariant model, in which the intercepts were set invariant in addition, did not yield an adequate fit to the data (χ^2^ = 363.85, *df* = 208, CFI = 0.79, TLI = 0.79, RMSEA = 0.07, SRMR = 0.14; Δχ^2^ = 104.76, Δ*df* = 15, *p* ≤ 0.001). Hence, mean comparisons across groups should only be made with caution.

### Total Score: Distribution and Differences Based on Background Variables (Studies 1–3)

**FIGURE 5:**
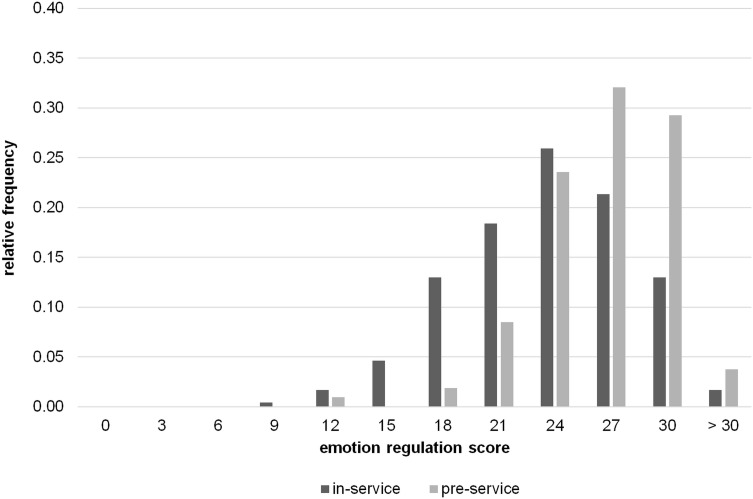
Distribution of the total scores in the *emotion regulation subtest* in the in-service (Study 1+2) and pre-service (Study 3) teacher samples.

**FIGURE 6:**
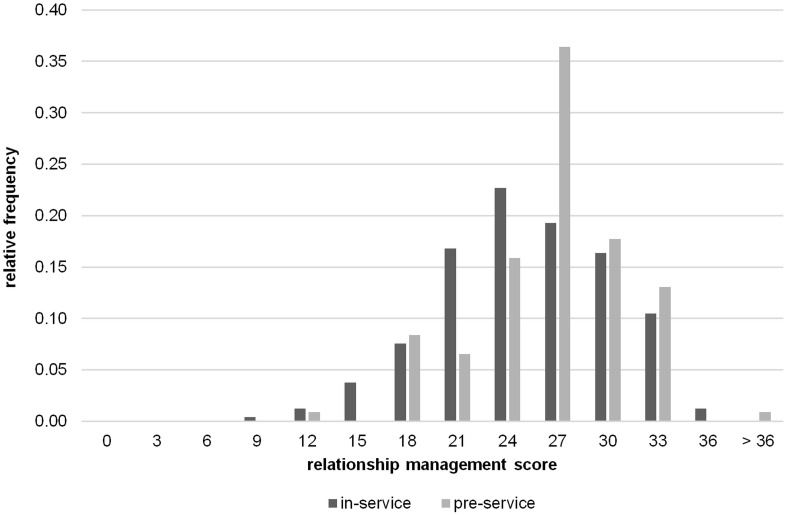
Distribution of the total scores in the *relationship management* subtest in the in-service (Study 1+2) and pre-service (Study 3) teacher samples.

We considered the computation of total scores for each subtest appropriate based on the satisfactory reliabilities for each subtest from the TRUST and results from factor analyses supporting a two-factor solution. The total score for the emotion regulation subtest was on average *M* = 22.29 in the in-service teacher samples and *M* = 25.19 in the pre-service teacher sample (theoretical maximum: 33 points). For the relationship management subtest, the total score was *M* = 23.90 in the in-service teacher samples and *M* = 25.33 in the pre-service teacher sample (theoretical maximum: 38 points). Hence, our participants’ social-emotional competence was, on average, fair. The distribution of the total scores is illustrated in Figures 5, 6.

As results from additional analyses showed (for detailed results see Supplementary Table A3), pre-service teachers obtained statistically significantly higher scores in the emotion regulation subtest than the in-service teachers [*F*(2,344) = 17.29, *p* < 0.001]. We also found statistically significant differences in relationship management scores depending on teachers’ experience level [*F*(2, 344) = 3.25, *p* = 0.040], but Scheffé *post hoc* tests did not reveal any specific group effects between pre- and in-service teachers. Within the group of in-service teachers, we did not find a statistically significant correlation between years of job experience and their emotion regulation (*r* = 0.02, *p* = 0.744) or relationship management scores (*r* = 0.04, *p* = 0.596). Finally, female teachers scored higher than male teachers in the relationship management [*t*(339) = –3.76, *p* < 0.001], but not in the emotion regulation subtest [*t*(339) = −1.51, *p* = 0.133]. Moreover, there was no statistically significant difference between in-service teachers working in academic- versus non-academic track schools [emotion regulation: *t*(237) = 1.88, *p* = 0.061; relationship management: *t*(236) = 1.22, *p* = 0.225].

### Convergent and Discriminant Validities (Study 3)

Our next goal was to investigate whether TRUST could be embedded in a nomological network of established and conceptually related constructs. More precisely, we aimed to examine whether TRUST was positively associated with pre-service teachers’ emotional intelligence (= convergent validity) and distinct from the Big Five personality traits showing at the most moderate correlations (= discriminant validity). As Table 2 displays, the TRUST emotion regulation subtest was positively and statistically significantly correlated with both the MSCEIT emotion management (*r* = 0.34, *p* = 0.001) and the MSCEIT emotional relationships (*r* = 0.28, *p* = 0.014) subtests. Likewise, the TRUST relationship management subtest was positively and statistically significantly correlated with MSCEIT emotion management (*r* = 0.23, *p* = 0.004) and MSCEIT emotional relationships (*r* = 0.30, *p* < 0.001) scores.

**TABLE 2:** Convergent and discriminant validities: correlation of the TRUST subtests with pre-service teachers’ (Study 3) emotional intelligence and personality.

	*M* (*SD*)	Emotion regulation	Relationship management
**TRUST**			
Emotion regulation	25.19 (3.49)		**0.45**
Relationship management	25.33 (4.45)		
**Emotional intelligence**			
Emotion management	37.45 (5.05)	**0.34**	**0.23**
Emotional relationships	47.53 (7.60)	**0.28**	**0.30**
**Personality**			
Agreeableness	3.28 (0.76)	0.17	**0.28**
Conscientiousness	3.74 (0.89)	0.16	**0.28**
Extraversion	3.82 (0.70)	0.05	0.05
Neuroticism	3.04 (1.08)	–0.01	–0.04
Openness	4.00 (0.74)	**0.21**	0.18

*Statistically significant coefficients at *p* < 0.05 are in bold.*

Regarding the association between TRUST scores and the Big Five personality traits, we found a statistically significant correlation between the emotion regulation subtest and pre-service teachers’ openness (*r* = 0.21, *p* = 0.012). Furthermore, relationship management scores yielded statistically significant associations with pre-service teachers’ agreeableness (*r* = 0.28, *p* = 0.001) and conscientiousness (*r* = 0.28, *p* = 0.006).

### Criterion Validity (Studies 1+2)

Finally, we aimed to investigate whether TRUST scores predicted in-service teachers’ occupational well-being and their self-reported quality of teacher-student interactions (see Table 3). Results showed a statistically significant positive correlation of emotion regulation scores and job satisfaction (*r* = 0.14, *p* = 0.037). Furthermore, teachers with more emotion regulation (*r* = −0.23, *p* = 0.009) and relationship management skills (*r* = −0.20, *p* = 0.013) reported fewer symptoms of depersonalization, which is one aspect of burnout. In contrast, we did not find statistically significant correlations between TRUST and teachers’ emotional exhaustion.

**TABLE 3:** Criterion validity: correlation of the TRUST subtests with in-service teachers’ (Study 1+2) occupational well-being and self-reported quality of social teacher-student interactions.

	*M* (*SD*)	Emotion regulation	Relationship management
**TRUST**			
Emotion regulation	22.29 (4.51)		**0.57**
Relationship management	23.90 (5.12)		
**Teacher-student interaction** (*N* = 91)			
Relationship	3.28 (0.40)	0.19	**0.27**
Emotional support	3.60 (0.31)	**0.43**	**0.42**
Behavior management	3.08 (0.56)	0.09	0.09
**Teacher well-being** (*N* = 239)			
Emotional exhaustion	2.45 (1.04)^1^	−0.14	−0.11
	2.21 (0.62)^2^		
Depersonalization	1.39 (0.83)^1^	−**0.23**	−**0.20**
	1.67 (0.65)^2^		
Job satisfaction	3.21 (0.66)	**0.14**	0.11

*^1^Scale ranging from 1 = *never* to 7 = *every day*; ^2^scale ranging from 1 = *disagree* to 4 = *agree;* statistically significant coefficients at *p* < 0.05 are in bold.*

To examine the link between TRUST and the quality of teacher-student interactions, we asked a subsample of in-service teachers (*n* = 91) about their individual perceptions of their relationship with students, the emotional support they provide, and their effectiveness in behavior management. Teachers with higher scores in the TRUST reported providing their students with more emotional support (emotion regulation: *r* = 0.43, *p* < 0.001; relationship management: *r* = 0.42, *p* < 0.001). In addition, teachers with better relationship management skills experienced a more positive relationship with their students (*r* = 0.27, *p* = 0.018). However, there was no statistically significant association between the TRUST subtests and the amount of classroom disturbances.

Finally, we investigated whether the results for convergent, discriminant, and criterion validities were stable when controlling for teachers’ age and gender. Statistically significant correlations remained identical. Furthermore, including these covariates, scores on the emotion regulation subtest were positively linked to the quality of teacher-student relationships (*r* = 0.19, *p* = 0.034).

## Discussion

The relevance of teachers’ social-emotional competence for the quality of teacher-student relationships, teacher well-being, and students’ development has been strongly emphasized from a theoretical perspective for over a decade (Brackett et al., 2006; Jennings and Greenberg, 2009). Despite a tremendous interest in teachers’ social-emotional competence, which includes their knowledge and skills required for mastering the social and emotional demands of their profession (Elias et al., 1997), empirical research in this field is, in our view, still constricted by a lack of objective and profession-specific measures. Therefore, our goal was to develop a theory-based situational judgment test of teachers’ social-emotional competence, more specifically, of their emotion regulation and relationship management skills. We hoped this tool would allow rigorous research in the field and, in addition, be useful for teacher education and professional development by providing the opportunity to assess teachers’ strengths in the social-emotional domain and help them learn about strategies for improving their emotion regulation and relationship management.

Results from three empirical studies with pre- and in-service teachers showed that the TRUST measured teachers’ social-emotional competence reliably. Confirmatory factor analyses supported its two-factor structure with one factor including the scenarios aiming to assess emotion regulation skills and the other one reflecting scenarios on relationship management skills. Regarding convergent validity, both subtests were statistically significantly and positively correlated with pre-service teachers’ general emotional intelligence. The finding that there were only small-to-moderate associations between TRUST scores and the Big Five personality traits showed that our tool measures more than personality and provides initial evidence for its discriminant validity. Moreover, in-service teachers with more emotion regulation and relationship management skills reported providing their students with more emotional support and having a better relationship with them. There was also a correlation between TRUST and symptoms of depersonalization, which is one symptom of burnout, but no link with teachers’ emotional exhaustion. Furthermore, we found a small positive association between higher emotion regulation scores and in-service teachers’ job satisfaction.

### Test Development, Item Characteristics, and Reliability of the TRUST

TRUST is composed of descriptions of short scenarios where teachers are confronted with emotional and social challenges in their interaction with students. For each scenario, we present four potential reactions and ask participants to rate the effectiveness of these reactions for regulating their own emotions (final version: seven scenarios) or for establishing and maintaining a positive teacher-student relationship (final version: eight scenarios). The scenarios were derived from Frenzel’s model of teacher emotions (Frenzel, 2014) and from prior research (e.g., Schmidt et al., 2017). The reactions reflect strategies that could be classified as differentially effective based on prominent theoretical frameworks on emotion regulation (Gross, 1998) and teacher-student interactions (Hamre and Pianta, 2007). The appropriateness of the scenarios, response choices, and coding scheme were tested in preliminary studies with in-service teachers and an expert sample of school psychologists. Hence, TRUST has a strong theoretical basis, but at the same time, we ensured to verify our theoretical ideas empirically.

Based on the three main studies we presented in this contribution, we were able to select a set of items (i.e., potential reactions to each scenario) that can distinguish between participants with higher and lower social-emotional competence. After item selection, the two subtests showed mostly satisfactory reliabilities in terms of Cronbach’s α. This was particularly remarkable because low internal consistencies are a common issue in situational judgment tests (Catano et al., 2012; Gold and Holodynski, 2015). As Lievens (2017) points out, most situational judgment tests in the past were designed to measure several different traits at the same time, which experts consider important to master professional tasks. This provides a threat to the unidimensionality of the measure. Lievens (2017) suggests that the construct-driven development of situational judgment tests could offer a solution. Our results support this claim and show that the construction of situational judgment tests with a pre-defined theoretical construct in mind, which is considered relevant for performance in critical professional situations, is a promising approach.

However, it is also important to note that the item-total correlations and reliabilities were lower in the pre-service than in the in-service teacher samples. Additional studies are needed to understand whether this variation is systematic in a way that teachers’ level of practical experience determines their interpretation of the reactions and their effectiveness evaluations or rather due to random factors. A promising research design for addressing this question would be a longitudinal study where participants report on the TRUST before finishing the university phase of their teacher education program and later, as in-service teachers. This design would also help to explain our counterintuitive finding that pre-service teachers received higher scores on the TRUST than in-service teachers did. We suggest that this is a cohort effect because universities in Germany are increasingly striving to integrate pedagogical-psychological contents in their teacher education programs (Hohenstein et al., 2014; Carstensen et al., 2019). Hence, our pre-service teachers may have profited from these learning opportunities.

### Two Subtests? The Factor Structure of the TRUST

Theoretically, emotion regulation and relationship management have been suggested as two distinct components of the overarching social-emotional competence construct (Zins et al., 2004). Therefore, we expected the TRUST subtests to be correlated, yet distinguishable. Results from confirmatory factor analyses largely supported this assumption, that is, two factors representing emotion regulation and relationship management skills emerged. The two factors were correlated substantially, but a one-factor solution was clearly inferior to a model with two distinct factors. Thus, it is appropriate to calculate a score for each subtest, which will allow future research to investigate whether emotion regulation and relationship management skills play differential roles in predicting various student and teacher outcomes.

However, we would also like to point out that one scenario from the emotion regulation subtest and two situations from the relationship management subtest had loadings that were rather small (λ < 0.30) in the pre-service teacher sample. This result was in line with the lower item-total correlations among pre-service teachers, which we have discussed in the previous paragraph. Perhaps, the low factor loadings were due to the content of the situations because the problems are more clearly attributable to the teacher rather than to student behavior. For example, in one scenario, students feel unfairly treated and, in another situation, a beginning teacher struggles in designing engaging lessons. Because teacher education hardly prepares teachers for dealing with disappointment and one’s own shortcomings, they may have to acquire this knowledge through practical experience. Consequently, responses to these scenarios may be distorted as they reflect pre-service teachers’ level of social-emotional competence, and in addition, whether they have encountered similar situations during internships.

### Capturing the Intended Construct? Convergent and Discriminant Validities of the TRUST

Having established appropriate measurement properties of the TRUST, our next goal was to provide initial evidence for its construct validity. First, we found support for convergent validity by establishing a positive and statistically significant association with pre-service teachers’ emotional intelligence. The correlations were moderate in size, which was in line with prior research investigating convergent validity between different measures of emotional intelligence (MacCann and Roberts, 2008; Austin, 2010). Furthermore, the moderate correlation between our profession-specific measure of social-emotional competence and a general emotional intelligence test is a first indicator regarding the value of our context-sensitive approach. To provide further evidence for this idea, future studies would profit from testing the incremental validity of TRUST beyond general emotional intelligence tests in predicting the quality of teacher-student interactions, student outcomes, and teacher well-being (e.g., a design combining and extending our Study 1 and Study 3).

Second, we aimed to ensure that TRUST was distinct from general personality traits. In line with this, we found small to moderate correlations with the Big Five. Teachers with higher scores in the emotion regulation subtest also had higher openness, which was a finding in line with research in emotional intelligence (Rossen and Kranzler, 2009). Considering the definition of openness as curiosity, wide-interest, and insightfulness, it is reasonable to assume that these characteristics increase people’s reflection on their emotions (McCrae and John, 1992; Schutte et al., 1998). Furthermore, agreeable and conscientious pre-service teachers obtained higher scores in the relationship management subtest. It seems plausible that teachers who have a tendency to be kind, sympathetic, and appreciative are better able to find solutions that meet students’ needs and, hence, help establish and maintain positive relationships. In line with this assumption, agreeable persons tend to have stronger interpersonal relationships (Asendorpf and Wilpers, 1998). Moreover, the correlation between conscientiousness and relationship management can be explained against the background that conscientiousness increases the likelihood of availing oneself of learning opportunities and taking professional responsibilities, such as building positive teacher-student relationships, seriously (Barrick and Mount, 1991).

Finally, we would also like to discuss the non-significant correlation with neuroticism because one might assume that people who are emotionally unstable and often worried should be more likely to ruminate or feel overwhelmed by negative emotions and, hence, unable to use adaptive strategies (John and Gross, 2004; Joseph and Newman, 2010). Furthermore, their emotional instability and touchiness could result in less effective relationship management (Neyer and Asendorpf, 2001; Deventer et al., 2019). However, TRUST asked participants to evaluate the effectiveness of different strategies. Thus, even though they may react differently in their daily lives, it is possible that neurotic people know that rumination, for example, is not an adaptive way of dealing with their emotions. Altogether, these results provide initial evidence that TRUST is associated with established concepts in expected ways, but still measures a unique construct that is distinct from general emotional intelligence and personality traits.

### Predictive for Outcomes in the “Real World”? Criterion Validity of the TRUST

#### Correlation With Occupational Well-Being

Based on the idea that adaptive emotion regulation helps people deal with negative emotions (Gross and John, 2003) and considering that social-emotional competence could reduce stressors and increase positive experiences in teachers’ interactions with students (Jennings and Greenberg, 2009), we expected a positive link between TRUST and in-service teachers’ occupational well-being in terms of high job satisfaction on the one hand and low emotional exhaustion and depersonalization on the other hand. In support of this, teachers with higher TRUST scores reported fewer symptoms of depersonalization, meaning that they were less prone to treating their students impersonally. Furthermore, there was a positive link between teachers’ emotion regulation skills and their job satisfaction. Contrary to our assumption, TRUST was not associated with emotional exhaustion. One explanation for this unexpected result could lie in the fact that we only focused on teachers’ strategies in dealing with challenges in their interactions with students. However, their profession includes many other, potentially stressful tasks as well (Kyriacou, 2011; Schmidt et al., 2017). Stressors that may cause emotional exhaustion frequently come from outside the classroom, for example, lesson preparation or organizational factors, making the competence aspects we measured less relevant (Aldrup et al., 2017). In future research it may be interesting to include measures more proximal to the contents of the TRUST, such as the Teacher Emotions Scale (Frenzel et al., 2016), which assesses teachers’ enjoyment, anger, and anxiety with regard to teaching.

#### Correlation With the Quality of Teacher-Student Interactions

Drawing on the ideas, for instance, of Jennings and Greenberg (2009) and preliminary empirical evidence (e.g., Voss et al., 2011; Jennings et al., 2017), we hypothesized that teachers with better emotion regulation and relationship management skills would be more successful in their interactions with students. Our findings were largely in line with this assumption and revealed that teachers who scored higher in the TRUST perceived closer relationships with students and reported providing more emotional support. In particular, the link between the relationship management subtest and emotional support stood out. This implies that teachers who know about strategies for establishing a positive climate, recognize students’ emotional, academic, and behavioral needs, and are able to differentiate between more and less appropriate approaches for responding to these needs, might behave correspondingly in their everyday teaching. That is, they indicate to provide additional support when needed, to listen to students’ opinions, and to treat them fairly. The somewhat less pronounced link with the quality of the teacher-student relationship reflects that the relationship is not only a function of teachers’ interpersonal behavior, but also of students’ prerequisites and reactions (Pianta et al., 2003; Nurmi and Kiuru, 2015). In other words, teachers’ social-emotional competence increases the likelihood that students will like them and turn to them when facing personal problems. Nonetheless, whether students feel connected to the teacher also depends on other factors, such as their relationship history with other teachers (Howes and Hamilton, 1992; McGrath and van Bergen, 2015). In contrast to the promising results for TRUST’s correlation with relationship quality and emotional support, we did not find a statistically significant link with behavior management. One explanation for this could be the fact that only a few scenarios asked teachers to deal with behavioral issues (one situation in the emotion regulation subtest, three situations in the relationship management subtest). To solve this issue, a revised and more comprehensive version of the test may profit from including additional scenarios, in which teachers must respond to students’ tardiness, disturbances, or need to re-establish rules. Alternatively, researchers who are particularly interested in teachers’ knowledge about behavior management and less so in their relationship management as a whole may use existing tests of general pedagogical-psychological knowledge (König et al., 2011; Voss et al., 2011) or strategic classroom management knowledge (Gold and Holodynski, 2015).

Regardless of these initial promising findings, we want to point out that both subtests showed similar patterns^1^ of correlations with teachers’ occupational well-being and their self-reported quality of teacher-student interactions, but also with emotional intelligence and personality. Even though this is logical considering that both are components of the higher-order social-emotional competence construct, future studies should investigate whether it is reasonable to distinguish between emotion regulation and relationship management, whether they yield differential associations with outcomes, and how they interact. In addition to assessing the overall quality of teacher-student interactions, it could be worthwhile to focus on performance in specific situations that can be hypothesized to depend more on emotion regulation or relationship management skills. For instance, how well teachers get to know a new group of friendly, curious students should depend on relationship management skills in particular, and has few demands about teachers’ emotion regulation.

### Limitations

In developing a situational judgment test that takes a profession-specific approach for measuring social-emotional competence in teachers, we provide an innovative tool for the research field. Nonetheless, the studies presented in the contribution can only be a starting point and additional research is needed to provide further validity evidence for the TRUST.

First, research with additional and larger samples would be needed to replicate the findings we obtained for the reliability and validity of the TRUST. Based on a sufficient sample size, it would also be possible to conduct factor analyses including the individual reactions to each scenario rather than parcels. This would allow for a more rigorous test of the factor structure.

Second, the correlations we found between TRUST and the quality of teacher-student interactions were based on teacher self-report measures. Whereas teacher ratings converge substantially with students’ or observers’ views on behavior management, teachers agree to a lesser degree with students on the quality of emotional support and the teacher-student relationship (Hughes and Kwok, 2007; Wagner et al., 2016; Aldrup et al., 2018a). Thus, examining whether TRUST scores predict student or observer ratings of interaction quality is an important next step.

Third, we aimed to include scenarios representing the various themes of daily teacher-student interactions, that is, interactions about students’ motivation, social-emotional or academic problems, as well as situations in which the teacher-student relationship *per se* was the focus (Frenzel, 2014; de Ruiter et al., 2019). However, these themes are not evenly represented. Thus, in further refining the TRUST one could aim to achieve a balance of themes in the scenarios. Including a sufficient number of situations for each theme in a more extensive version could also be insightful for understanding whether individual teachers perform equally well independent of the theme or whether they have strengths and weaknesses in specific areas.

Finally, the scenarios concentrate on students as interaction partners, but teachers face emotional and social challenges in their interactions with colleagues or parents as well (Pyhältö et al., 2011; Schmidt et al., 2017). We think our focus is justified because students are not only teachers’ most frequent interaction partner, but high-quality teacher-student interactions are also a key prerequisite for student development and, hence, at the core of teachers’ professional responsibilities (Pianta and Hamre, 2009). Nonetheless, researchers interested in the whole range of teachers’ social and emotional lives should consider the specific content of the TRUST scenarios. Moreover, the scenarios are situated at the secondary school level, potentially making the test more difficult and less engaging for elementary school teachers.

## Conclusion and Implications

Our results provide satisfactory evidence for the reliability and validity of the TRUST in capturing teachers’ emotion regulation and relationship management skills. Therefore, it is a promising tool for the thriving research field on the social and emotional aspects of the teaching profession (e.g., Uitto et al., 2015; Klingbeil and Renshaw, 2018). On the one hand, it could be used to empirically test the theoretical model suggested by Jennings and Greenberg (2009), to see how the different facets of social-emotional competence are linked to the quality of emotional support and behavior management, student outcomes, or teacher well-being. On the other hand, TRUST could be used to evaluate teacher education and professional development courses. Moreover, it could be integrated in these courses and for informal self-reflection. Thinking about the potential reactions included in the test could make teachers more conscious of their behavior in emotionally and socially challenging situations and may help to discover alternative approaches they would not have considered before. Furthermore, teacher educators could discuss the advantages and drawbacks as well as short- and long-term consequences of different reactions to a given situation. Altogether, we hope that the development of TRUST will contribute to a more profound and empirically supported understanding of the role of teachers’ social-emotional competence in the development of both students and teachers. Ultimately, these insights are key for informing decisions about the content of teacher education and professional development programs.

## Data Availability Statement

The datasets generated for this study are available on request to the corresponding author.

## Ethics Statement

Ethical review and approval was not required for the study on human participants in accordance with the local legislation and institutional requirements. The patients/participants provided their written informed consent to participate in this study.

## Author Contributions

All authors were involved in test development and data collection. KA conducted the statistical analyses and wrote the manuscript. BC, MK, and UK provided feedback during the whole process.

## Conflict of Interest

The authors declare that the research was conducted in the absence of any commercial or financial relationships that could be construed as a potential conflict of interest.

## Footnotes

1 We tested whether the correlations with the MSCEIT, personality, interaction quality, and well-being were invariant across the *emotion regulation* and the *relationship management* subtests. This was the case. Hence, there were no statistically significant differences in the correlations.
