# Supplementary material for: Measuring Teachers’ Social-Emotional Competence: Development and Validation of a Situational Judgment Test
Source: Front Psychol. 2020 May 29;11:892. doi: 10.3389/fpsyg.2020.00892 (PMC7273885; doi:10.3389/fpsyg.2020.00892)
Supplement: Supplementary file 1 [file Table_1.PDF]

Supplemental Material

Measuring Teachers' Social-Emotional Competence: Development and Validation of a Situational Judgment Test

This online supplement provides an overview of the results from the expert study with *N* = 13 school psychologists (Table A1, Table A2) as well as results for additional analyses on differences in test scores based on background variables (Table A3).

In the left part of Table A1 and Table A2, you see how effective experts rated the *very effective* and the *very ineffective* strategies on average. Moreover, we display the percentage of experts who selected the exact correct response (1 point) and the percentage of experts who selected the correct or at least a partially correct response (≥ ½ points). In the right part of Tables A1 and A2, you see the results for the pairwise comparisons between the *ambiguous* strategies and the adjacent/distant very (in-)effective strategies. On the one hand, you can see the average difference between the two strategies. On the other hand, we summarize the percentage of experts who correctly differentiated between the two strategies. We would like to give an example for reading the tables in the following.

In Table A1 you see that the very ineffective strategy B1_2 was rated, on average, rather low by the experts (*M* = 1.54). The exact correct response of 1 = *very ineffective* was selected by 46.15% of the school psychologists. As indicated by the fact that 100.00% of the experts received ½ point or more, there was no one who rated this item higher than 2 = *slightly ineffective*. In the right part of Table A1, you can see the pairwise comparison between B1_2 and the ambiguous strategy B1_1. We considered B1_1 in tendency effective so that the effectiveness of this ambiguous strategy is rather distant from the very ineffective strategy B1_2. On average, experts considered B1_1 and B1_2 quite different (*M* = 2.15 units apart). Because we classified these strategies as distant regarding their effectiveness, respondents received 1 point for scoring the strategies at least 2 units apart. This was the case for 69.23% of the experts. As indicated by the fact that 100% of the experts received ½ point or more, all school psychologists considered B1_1 more effective than B1_2.

Table A1

Experts' Responses to the Emotion Regulation *Subtest: In the Left Part are the Mean Effectiveness Ratings for the* Very Effective *and* Very Ineffective *Strategies and the Percentage of Experts Who Received 1 Point or at Least ½ Point. In the Right Part are the Mean Differences Experts Perceived Between the* Ambiguous *and the* Very (In-)Effective *Strategies and Points Obtained for the Pairwise Comparisons.*

**Very Effective Strategies**				**Very Ineffective Strategies**				**Pairwise Comparisons of Adjacent Strategies**				**Pairwise Comparisons of Distant Strategies**			
	M	**1P**	**≥ ½P**		***M***	**1P**	**≥ ½P**		***M*[1]**	**1P**	**≥ ½P**		***M*[1]**	**1P**	**≥ ½P**
B1_3	4.46	69.23%	92.31%	B1_2	1.54	46.15%	100.00%	B1_13	0.77	69.23%	84.62%	B1_12	2.15	69.23%	100.00%
B2_1	4.77	76.92%	100.00%	B3_3	1.85	30.77%	84.62%	B1_34	0.46	61.54%	84.62%	B1_24	2.46	84.62%	92.31%
B2_4	4.54	61.54%	92.31%	B5_4	1.85	30.77%	92.31%	B2_13	1.23	76.92%	100.00%	B2_12	2.23	84.62%	92.31%
B3_4	4.85	84.62%	100.00%	B6_3	1.38	61.54%	100.00%	B2_34	1.00	76.92%	100.00%	B2_24	2.00	84.62%	84.62%
B4_1	4.46	46.15%	100.00%	B7_4	1.62	46.15%	92.31%	B3_14	1.38	84.62%	100.00%	B3_13	1.62	61.54%	84.62%
B4_3	4.23	46.15%	84.62%	B8_1	1.46	53.85%	100.00%	B3_24	1.23	76.92%	100.00%	B3_23	1.77	61.54%	84.62%
B5_1	4.69	76.92%	92.31%					B4_12	0.54	53.85%	84.62%	B4_14	1.85	61.54%	92.31%
B5_3	4.54	61.54%	92.31%					B4_23	0.31	38.46%	76.92%	B4_34	1.62	46.15%	92.31%
B6_1	4.38	61.54%	84.62%					B5_12	1.15	69.23%	92.31%	B5_24	1.69	61.54%	76.92%
B7_2	4.54	53.85%	100.00%					B5_23	1.00	76.92%	100.00%	B6_23	1.85	61.54%	84.62%
B7_3	4.54	53.85%	100.00%					B6_12	1.15	61.54%	84.62%	B6_34	2.38	76.92%	92.31%
B8_2	4.23	30.77%	92.31%					B6_14	0.62	38.46%	84.62%	B7_12	2.00	76.92%	100.00%
								B7_14	0.92	61.54%	92.31%	B7_13	2.00	69.23%	100.00%
								B8_13	1.31	84.62%	100.00%	B8_12	2.77	100.00%	100.00%
												B8_14	2.54	92.31%	100.00%

Note. [1]*Mean difference, which is calculated as follows: difference = more effective strategy – less effective strategy; P = point; items and pairwise comparisons that were excluded in the final test version due to low item-total correlations are in grey (dark grey = pairwise comparisons from scenarios that were retained, light grey = items and pairwise comparisons from scenarios that were completely excluded).*

Table A2

Experts' Responses to the Relationship Management *Subtest: In the Left Part are the Mean Effectiveness Ratings for the* Very Effective *and* Very Ineffective *Strategies and the Percentage of Experts Who Received 1 Point or at Least ½ Point. In the Right Part are the Mean Differences Experts Perceived Between the* Ambiguous *and the* Very (In-)Effective *Strategies and Points Obtained for the Pairwise Comparisons.*

**Very Effective Strategies**				**Very Ineffective Strategies**				**Pairwise Comparisons of Adjacent Strategies**				**Pairwise Comparisons of Distant Strategies**			
	M	**1P**	**≥ ½P**		***M***	**1P**	**≥ ½P**		***M*[1]**	**1P**	**≥ ½P**		***M*[1]**	**1P**	**≥ ½P**
C1_3	4.08	46.15%	84.62%	C1_2	2.46	7.69%	69.23%	C1_13	0.08	38.46%	69.23%	C1_12	1.54	46.15%	69.23%
C2_3	4.54	53.85%	100.00%	C2_2	1.54	53.85%	92.31%	C1_34	0.17	50.00%	75.00%	C1_24	1.42	41.67%	83.33%
C4_1	4.75	75.00%	100.00%	C2_4	1.31	76.92%	92.31%	C2_12	2.46	100.00%	100.00%	C2_13	0.54	0.00%	53.85%
C5_1	4.50	58.33%	91.67%	C3_3	2.50	8.33%	58.33%	C2_14	2.69	100.00%	100.00%	C3_23	0.92	33.33%	66.67%
C6_4	4.67	66.67%	100.00%	C3_4	2.67	8.33%	41.67%	C3_13	1.50	83.33%	91.67%	C3_24	0.75	41.67%	58.33%
C7_2	4.73	72.73%	100.00%	C4_3	2.50	8.33%	58.33%	C3_14	1.33	75.00%	100.00%	C4_23	0.50	25.00%	41.67%
C7_4	4.67	66.67%	100.00%	C4_4	1.50	66.67%	91.67%	C4_12	1.75	83.33%	100.00%	C4_24	1.50	41.67%	83.33%
C8_2	4.58	58.33%	100.00%	C5_2	1.50	66.67%	91.67%	C5_13	0.92	75.00%	83.33%	C5_23	2.08	75.00%	91.67%
C9_1	4.75	75.00%	100.00%	C5_4	1.50	66.67%	83.33%	C6_12	1.25	75.00%	83.33%	C5_34	2.08	66.67%	100.00%
C9_4	4.92	91.67%	100.00%	C6_1	1.58	50.00%	91.67%	C6_34	0.58	41.67%	91.67%	C6_13	2.50	75.00%	91.67%
				C7_1	1.50	50.00%	100.00%	C7_13	0.92	58.33%	83.33%	C6_24	1.83	41.67%	91.67%
				C8_1	1.83	33.33%	83.33%	C8_13	1.42	75.00%	100.00%	C7_23	2.18	72.73%	81.82%
				C8_4	1.73	36.36%	90.91%	C8_34	1.55	81.82%	100.00%	C7_34	2.25	75.00%	75.00%
								C9_12	1.08	66.67%	91.67%	C8_23	1.33	41.67%	75.00%
								C9_13	1.33	75.00%	91.67%				
								C9_24	1.25	66.67%	100.00%				
								C9_34	1.50	75.00%	100.00%				

Note. [1]*Mean difference, which is calculated as follows: difference = more effective strategy – less effective strategy; P = point; items and pairwise comparisons that were excluded in the final test version due to low item-total correlations are in grey (dark grey = pairwise comparisons from scenarios that were retained, light grey = items and pairwise comparisons from scenarios that were completely excluded).*

Table A3

Total Scores in the Emotion Regulation *and* Relationship Management *Subtests Depending on Background Variables*

	**Gender** *(M, SD)*			**Career Status**[1] *(M, SD)*				**School Type**[1] *(M, SD)*		
	Male	Female	*t(df)*	Bachelor Student	Master Student	In-Service Teacher	*F(df)*	Academic	Non-Academic	*t(df)*
n	106	235		64	42	236		120	119	
**Emotion Regulation**	22.65	23.43	–1.51 (339)	25.09_a	25.36_a	22.29_b	**17.29** (2, 344)	22.84	21.74	1.88 (237)
**Relationship Management**	22.83	24.97	**–3.76** (339)	25.12	25.65	23.90	**3.25** (2, 344)	24.31	23.50	1.22 (236)

Note. [1]Results remained the same in ANCOVAs with gender as covariate; statistically significant coefficients at $p < .05$ are in bold; means with different subscripts were statistically significantly different at $p < .05$ in ANOVA with Scheffé post-hoc tests.
